# Prosociality and a Sociosexual Hypothesis for the Evolution of Same-Sex Attraction in Humans

**DOI:** 10.3389/fpsyg.2019.02955

**Published:** 2020-01-16

**Authors:** Andrew B. Barron, Brian Hare

**Affiliations:** ^1^Department of Biological Sciences, Macquarie University, Sydney, NSW, Australia; ^2^Department of Evolutionary Anthropology, Center for Cognitive Science, Duke University, Durham, NC, United States

**Keywords:** self-domestication, homosexual, endocrine hypothesis, testosterone, sexual inversion, sexuality, bonobo

## Abstract

Human same-sex sexual attraction (SSSA) has long been considered to be an evolutionary puzzle. The trait is clearly biological: it is widespread and has a strong additive genetic basis, but how SSSA has evolved remains a subject of debate. Of itself, homosexual sexual behavior will not yield offspring, and consequently individuals expressing strong SSSA that are mostly or exclusively homosexual are presumed to have lower fitness and reproductive success. How then did the trait evolve, and how is it maintained in populations? Here we develop a novel argument for the evolution of SSSA that focuses on the likely adaptive social consequences of SSSA. We argue that same sex sexual attraction evolved as just one of a suite of traits responding to strong selection for ease of social integration or prosocial behavior. A strong driver of recent human behavioral evolution has been selection for reduced reactive aggression, increased social affiliation, social communication, and ease of social integration. In many prosocial mammals sex has adopted new social functions in contexts of social bonding, social reinforcement, appeasement, and play. We argue that for humans the social functions and benefits of sex apply to same-sex sexual behavior as well as heterosexual behavior. As a consequence we propose a degree of SSSA, was selected for in recent human evolution for its non-conceptive social benefits. We discuss how this hypothesis provides a better explanation for human sexual attractions and behavior than theories that invoke sexual inversion or single-locus genetic models.

In most contemporary human cultures that have been studied individuals who self-identify as exclusively homosexual are rare (Ward et al., 2014; Bailey et al., 2016), but a larger minority of the population report some homosexual sexual behavior and experience and a degree of same sex sexual attraction (SSSA) (Bagley and Tremblay, 1998; Savin-Williams and Vrangalova, 2013; Bailey et al., 2016). While estimates of the population prevalence and distribution of SSSA vary (Bailey et al., 2016) contemporary studies support Kinsey et al.’s (1948, 1953) conclusion that in human populations there is continuous variation in the expression of homosexuality. The variation forms a smooth cline from a large majority who report exclusive or mostly heterosexual attraction and/or behavior, through groups who report degrees of both homosexual and heterosexual attractions and/or behavior to a small minority who report exclusive homosexual attractions and behavior (Savin-Williams and Vrangalova, 2013; Bailey et al., 2016).

For evolutionary biologists SSSA and associated homosexual sexual orientation has presented somewhat of a conundrum. SSSA persists both within and across cultures (Witham and Mathy, 1985; Crompton, 2006) and within families, since sexual orientation has high heritability (Pillard and Bailey, 1998; Mustanski et al., 2005; Santtila et al., 2008; Bailey et al., 2016). Evidence from human twin studies and genome-wide genetic association studies suggest that about one third of the variation in sexual orientation can be attributed to additive genetic factors (Santtila et al., 2008; Bailey et al., 2016; Ganna et al., 2019). For evolutionary biologists the puzzle is typically posed like this: how can a heritable SSSA persist in a population when homosexual sex of itself is non-reproductive and homosexual people have fewer offspring on average than heterosexual people (Bell et al., 1981; King et al., 2005; Wrangham, 2019). There is expected to be strong selection against genetic factors that contribute to SSSA: how, therefore, can heritable homosexual attractions persist in a population (Kirkpatrick, 2000; Gavrilets and Rice, 2006; Bártová and Valentová, 2012; Rice et al., 2012; Jeffery, 2015)?

Various explanations to this puzzle have been proposed. The prevalence of SSSA is certainly too high for the trait to be maintained by recurrent random mutation (Moran, 1972). Models have consequently been proposed to explain how SSSA could be maintained in a population as a stable genetic polymorphism, but presently there is scant or no evidence to support these theories.

The theory of sexually antagonistic selection proposes that genetic factors contributing to SSSA in one sex could persist in populations if they conferred a strong selective advantage when expressed in the other sex, and various models of this kind have been posed to explain human SSSA (Gavrilets and Rice, 2006; Camperio-Ciani et al., 2008; Rice et al., 2012). Camperio-Ciani et al. (2008) explored whether the female relatives of homosexual males had more offspring than female relatives of heterosexual males, which could be indirect evidence for antagonistic sexual selection. There is some evidence that females with male homosexual relatives have more children than females with no male homosexual relatives in a Western European population (Camperio-Ciani et al., 2008; Lemmola and Camperio-Ciani, 2009), but this finding is at best only weakly supported in other populations or cultures (Vasey et al., 2007; Bailey et al., 2016; Semenyna et al., 2017), and so overall there is little evidence for sexually antagonistic selection as an explanation for SSSA in human males. No study has yet explored whether this theory might apply to human females.

An alternative hypothesis proposes that SSSA and homosexual behavior could be maintained in a population if genetic factors contributing to these traits had pleiotropic effects that conferred a reproductive advantage. Zietsch et al. (2008) explored a version of this hypothesis and reported that psychologically masculine females and psychologically feminine men typically identified as non-heterosexual, but if did self identify as heterosexual they also self-reported a greater number of sexual partners than average for heterosexuals. We note that number of sexual partners is a long way from a measure of reproductive success or fitness. We also note that a critical test of this hypothesis was whether heterosexuals with a non-heterosexual identical twin have more sexual partners than members of heterosexual identical twin pairs. Here, there was a trend in the hypothesized direction but no statistically significant difference in number of sexual partners (Zietsch et al., 2008). Zietsch et al.’s (2008) study is certainly intriguing. The data they present are compatible with a relationship between SSSA, homosexual behavior and increased socio-sexuality – a point we develop later.

Kin-selection theories propose genes promoting SSSA could persist in a population if people expressing SSSA enhanced the reproduction of relatives (Bailey and Zuk, 2009). It is assumed the indirect fitness benefit of more relatives would compensate for the presumed fitness costs associated with SSSA and same-sex sexual behavior. Invoking kin-selection theory to explain human SSSA seems a little odd. The many examples of social and reproductive traits in animals that have evolved as a consequence of kin selection emphasize the evolution of non-reproductives, not same-sex sexual behavior (Kirkpatrick, 2000). In human societies there is very little evidence homosexual people increase the reproductive output of relatives (Bobrow and Bailey, 2001; Rahman and Hull, 2005; Vasey and VanderLaan, 2012; Abild et al., 2014; Prum, 2017) offering weak empirical support to kin selection theories for SSSA. But several studies by Vasey and others have emphasized the avuncularity (defined as altruistic uncle-like behavior) and generosity of transgender males expressing SSSA (Vasey et al., 2007; Vasey and VanderLaan, 2012, 2015; Abild et al., 2014) perhaps indicating a relationship between SSSA and affiliative behavior.

Here we propose a sociosexual hypothesis for the evolution of SSSA that explores possible adaptive social functions of same-sex directed attractions and behavior. Benefits of SSSA and same sex sexual behavior for the development and maintenance of same sex social bonds and group affiliation have been proposed previously, most notably by Kirkpatrick (2000) and later by Bártová and Valentová (2012). But here we link the evolution of human SSSA to the suite of traits that evolved as a consequence of selection for ease of social integration (prosocial behavior), within-group tolerance and social affiliation. This has been described as an evolutionary process of human self-domestication (Eisenberg et al., 1983; Clay and Zuberbühler, 2011; Gleeson, 2016; Hare, 2017; Theofanopoulou et al., 2017; Niego and Benítez-Burraco, 2019; Wrangham, 2019).

Assessing SSSA in non-human animals is not easy, but what is clear is that homosexual *behavior* is not a human innovation. It is widespread in primates (Sommer and Vasey, 2006) and other animals (Bagemihl, 1999; Bailey and Zuk, 2009), and is certainly ancestral to hominids. Analyses of the contexts of occurrence of homosexual sexual behavior in primate societies suggest the behavior has various diverse functions. These include appeasement, pacification, reinforcement of social dominance structures, juvenile play, social tolerance, stress reduction, and barter (Sommer and Vasey, 2006; Clay and de Waal, 2015). Heterosexual sexual behavior shows a similar diversity of expression across primate societies (Sommer and Vasey, 2006). It appears there has been an expansion of the social functions of sexual interactions (both homosexual and heterosexual) as more complex societies evolved in primates (Werner, 2006). As a consequence, sexual behavior in primates has been subject to selection for adaptive social functions as well as the obvious reproductive functions.

Social evolutionary processes have been a major driver of recent human cognition and behavior (Eisenberg et al., 1983; Dos Santos and West, 2005); particularly selection for increased intra-group tolerance and reduced intra-group aggression (Bowles and Gintis, 2013; Hare, 2017). Prosocial individuals would have more readily accessed the fitness benefits of cooperative group living (Hare, 2017), and would have gained both greater reproductive success and social mobility (Bowles and Gintis, 2013). Enhanced tolerance also would allow for smoother integration of juveniles that moved from their natal group to a new group – bringing new ideas and technology with them. Selection for prosociality is thought to have driven the recent evolution of bonobos from their chimp-like ancestor, and proto-dogs from their wolf-like ancestor also (Hare, 2017).

In humans, dogs, and bonobos, a common suite of traits has evolved as a consequence of selection for prosociality. These are juvenilization of facial features, extended cognitive developmental periods, reduced social threat responses, reduced aggression, reduced aggressive reactivity, cooperative play behavior, and increased cooperative-communicative capacity and engagement (Hare, 2017; Theofanopoulou et al., 2017; Wrangham, 2019). This set of traits is very similar to those that have arisen from artificial selection on species for reduced aggression and fear of humans in order to domesticate them (Belyaev et al., 1985; Hare, 2017). Consequently, recent human evolution has been described as a process of self-domestication arising from natural selection for prosocial behavior (Gleeson, 2016; Hare, 2017; Niego and Benítez-Burraco, 2019; Wrangham, 2019).

Across both domesticated species and self-domesticated species it is common to see an increase in expression of same-sex sexual behavior. This is part of the expansion of the contexts of sexual behavior (same-sex oriented and heterosexual) into adult play, usually interpreted as part of an adult affiliative function for sex (Dagg, 1984; Poiani, 2011). For example, in the evolution of dogs from wild dogs, and wild dogs from wolves both self-domestication and domestication have increased expression of adult sexual play and homosexual sexual behavior relative to their wild relatives (Dagg, 1984). While domestication of livestock has not always increased rates of homosexual behavior, there are several well studied examples where domestication has yielded high levels of same-sex sexual behavior among adults (Dagg, 1984; Perkins and Roselli, 2007).

The bonobo has experienced a parallel process to humans of prosocial evolution from a chimp-like ancestor (Hare, 2017; Tan et al., 2017). Like humans, bonobos show a suite of features associated with self-domestication (Hare, 2017). Bonobos exhibit higher levels than chimpanzees of same-sex sexual behavior in contexts of adult play and social affiliation also (Clay and Zuberbühler, 2011; Dixon, 2011; Woods and Hare, 2011; Bailey et al., 2016; Hare and Yamamoto, 2017).

Same-sex sexual attraction, homosexual behavior and same sex affiliations are distinct dimensions of sexuality (Bolin and Whelehan, 2009; Jordan-Young, 2010; Greenberg et al., 2016; Valentova and Varella, 2016), but they are related. SSSA is a motivator of homosexual behavior, and sexual behavior is a strong motivator of social bonds and affiliations. Sex is a strong reinforcer of pair bonds in all social mammals studied (Young and Wang, 2004). Sexual behavior in social contexts functions as a reinforcer of social bonds also (Kirkpatrick, 2000). Same-sex social bonds are likely to be as important as heterosexual social bonds for any individual operating within a social group (Kirkpatrick, 2000). A degree of SSSA could therefore reasonably confer a selective advantage, by facilitating engagement in sociosexual behavior with the associated benefits of social reinforcement, affiliation, play, appeasement, and conflict resolution (Kirkpatrick, 2000; Bártová and Valentová, 2012). Selective benefits for SSSA could be increased ease of social bonding and reduced intragroup conflict through a willingness to engage in or initiate homosexual sexual play. Human ethnographic evidence points to an adaptive benefit for SSSA in alliance formation and maintenance (Kirkpatrick, 2000; Muscarella et al., 2005).

Mechanistic analyses indicate links between increased prosociality and SSSA. Raghanti et al. (2018) have argued that the neurochemical profile of the human striatum is unique among primates with elevated dopamine, serotonin, and neuropeptide Y signaling. They argue this feature evolved early in hominid evolution and increased sensitivity to social cues to promote empathy and affiliative behavior (Raghanti et al., 2018). Self domestication in both dogs and humans is believed to have caused evolutionary changes in serotonin, oxytocin and androgen systems that regulate affiliative, threat, and aggressive behavior (Hare, 2017), and are involved in chimpanzee social affiliation (Samuni et al., 2017). These are the same endocrine systems that have been implicated in the development of human SSSA and homosexual behavior (Mustanski et al., 2002; Balthazart, 2011; Fleischman et al., 2015). In domesticated sheep changes in these neurochemical systems have been considered causal of increased levels of homosexual behavior (Perkins and Roselli, 2007). Taken together, these studies suggest an overlap between the neurochemical systems involved in affiliation and prosocial behavior and those involved in an increased incidence of same-sex sexual behavior in animals. Such a relationship is expected given that sex is itself a mechanism of social bonding in mammals (Young and Wang, 2004; Young et al., 2005).

Prosociality, increased in-group tolerance and increased social affiliation: these are extremely complex traits involving widespread changes in behavior, anatomy, and neurophysiology (Hare, 2017; Theofanopoulou et al., 2017; Raghanti et al., 2018). Genetic changes underlying the evolution of such traits are likely to be complex and highly polygenic. Presently not much is known about the genetic basis of human SSSA, but as we learn more about it, it is clear human SSSA is also highly polygenic and a complex multicomponent trait (Mustanski et al., 2005; Prum, 2017; Sanders et al., 2017; Ganna et al., 2019; Swift-Gallant et al., 2019). The high heritability of human SSSA is caused by a large number of genes each with individually small effect. These genes likely contribute to different aspects of sexuality which can assort independently (Mustanski et al., 2005; Sanders et al., 2017; Ganna et al., 2019). Genetic models for the evolution of human SSSA should therefore reflect this complexity and be polygenic and multicomponent, rather than positing individual genes of large effect, as has occurred previously (Gavrilets and Rice, 2006; Rice et al., 2012).

A polygenic and additive genetic model of SSSA is compatible with the nature and distribution of SSSA in human populations, which features continuous variation in the degree of SSSA from a majority reporting exclusively heterosexual attractions to a small minority reporting exclusively homosexual attractions (Bailey et al., 2016). Along this cline of variation individuals expressing degrees of both homosexual and heterosexual attractions are stable sexualities and not transitional forms (Bailey et al., 2000; Diamond, 2008; Rosenthal et al., 2012; Savin-Williams and Vrangalova, 2013). We propose this pattern of variation could have arisen from selection for prosociality increasing the frequency of alleles in a population across multiple loci that contribute to prosocial behavior. This would include alleles contributing to SSSA because of the benefits of sociosexual same-sex behavior for same-sex social bonding and affiliation. If a trait is highly polymorphic and polygenic [as sexual orientation seems to be (Sanders et al., 2017; Ganna et al., 2019)] the random recombination of genes in sexual reproduction would result in a spectrum of heritable variation for strength of SSSA in a population (Prum, 2017).

Given this argument one might ask why SSSA is not more common in human populations. Indeed, Kirkpatrick (2000) wondered that bisexuality might be an adaptive optimum since it would allow for sociosexual affiliative behavior with members of both sexes. Kirkpatrick (2000) proposes that any reproductive disadvantage from a low level of same-sex sexual behavior could be minor or negligible, irrespective of the degree of SSSA associated with the behavior.

To this point we note simply that while individuals reporting exclusive SSSA are rare in most contemporary human populations, SSSA is not. While specific measures vary all studies recognize that males and females reporting some degree of SSSA are relatively common, and not rare (Kinsey et al., 1948, 1953; Kirkpatrick, 2000; Mustanski et al., 2002; Bailey et al., 2016). Bisexuality is more common than homosexuality, but the nature of variation in SSSA is often not well appreciated since experimentalists are prone to force a binary dichotomy across what is in reality continuous and multivariate variation in sexuality (Jordan-Young, 2010). There may also be cultural reasons why the degree of SSSA in populations may go under-reported.

We emphasize that our hypothesis is not that homosexual people are domesticated, or even more prosocial than the population average. Rather, we recognize that self-domestication has been an important process in the recent evolution of our species as a whole. SSSA has increased in frequency in humans as a consequence of the self-domestication syndrome experienced by our species. If correct, this sociosexual hypothesis comprehends the phenomenon of human SSSA as part of broader adaptive prosocial changes in recent human cognitive and social evolution (Burkart et al., 2009, 2014; Hare, 2017).

Two other authors have remarked on a link between SSSA and selection for prosociality: Prum (2017) and Wrangham (2019). Prum (2017) argues that for humans female dispersal was the ancestral condition, with females rather than males leaving their natal group. He proposes that female SSSA could evolve as part of selection for female prosociality to aid female introgression into a new social group and strengthen female-female social bonds (Prum, 2017, p. 508). He further argues that male SSSA and homosexual behavior could have evolved through female mate choice (Prum, 2017). Females may have preferred males that show a degree of SSSA since this male trait would lessen the intensity and investment of males in sexual and social control of females, and would subsequently have fostered the evolution of prosocial males and more cooperative male–male and female–male relationships (Prum, 2017, p. 509).

Wrangham (2019) has also recognized an association between prosociality and homosexuality, but Wrangham proposes a very different hypothesis for why this association might be so. Wrangham (2019, p. 189) suggests human homosexuality is a maladaptive by product of selection against reactive aggression in humans. Wrangham (2019) argues that selection for reduced reactive aggression reduced prenatal testosterone levels in males, which resulted in a maladaptive expression of homosexuality in a minority of males.

Models of human evolution are naturally hard, if not impossible, to prove or disprove, but here we note that Wrangham’s explanation for an association between homosexuality and prosociality does not, and cannot, explain homosexuality in women. By contrast, prosocial benefits of SSSA would be expected to apply to both female–female social relationships and male–male social relationships (Kirkpatrick, 2000). Prum’s (2017) evolutionary argument is interesting in many ways, not least of which is because it proposes different (but interacting) selective pressures for the evolution of male and female SSSA in humans. Here we have argued a link between prosocial evolution and SSSA. Prum (2017) recognizes this selective force for females, but considers female mate choice the primary driver of human male SSSA, with prosociality in human males an outcome of female mate choice. This hypothesized evolutionary scenario is perhaps more complex than ours, but that does not mean it is less likely. If non-prosocial species could be found in which female mate choice had lead to the evolution of male SSSA this would lend strong support to Prum’s (2017) model for social evolution.

Wrangham’s reasoning and evidence draw on the endocrine hypothesis for human homosexuality, which has been strongly refuted (Jordan-Young, 2010). There are many variants of the endocrine hypothesis, but they all propose that SSSA is caused by some malfunction or gendered misexpression of endocrine systems considered responsible for establishing gender-typical behavioral differences between heterosexual males and heterosexual females (Mustanski et al., 2002; Balthazart, 2011; Rice et al., 2012; Bailey et al., 2016). Hypotheses vary as to when or how in development a change in endocrine systems could result in SSSA. Arguments in support of the endocrine hypothesis come from a range of experimental manipulations of mammals, including primates, which demonstrate a role for androgens in the organization and development of male and female typical sexual and social behavior, and also show that severe manipulations of endocrine systems in early development can result in males showing female-typical sexual behavior and vice-versa (Balthazart, 2011; Poiani, 2011).

The endocrine hypothesis does not, however, fit well to features of human SSSA (Jordan-Young, 2010). The example of female congenital adrenal hyperplasia (CAH) is often cited as evidence supporting an endocrine basis of human SSSA (Balthazart, 2011). This disorder causes prenatal hypertrophy of the adrenal gland, and consequently the developing fetus is exposed to higher than normal levels of testosterone. Females with CAH report a higher incidence of adult homosexual orientation than that of the population as a whole, but most females with CAH report exclusively heterosexual attraction (Meyer-Bahlburg et al., 2008; Jordan-Young, 2010). This would suggest that for women there is not a simple relationship between elevated prenatal testosterone and SSSA. Further, in both animal studies and the human cases of CAH pre- or perinatal endocrine manipulations have consequences for the development of anatomical secondary sexual characteristics and genital morphology. Female rhesus monkeys given testosterone postnatally develop an enlarged clitoris (Pfaff, 1999; Dixson, 2013) and some females with CAH also develop partially masculinized genitalia (Bailey et al., 2016). There is no evidence that homosexual people (male or female) have intersex genital development (Jordan-Young, 2010; Bailey et al., 2016) suggesting it is unlikely an endocrine imbalance pre or perinatally is causal of human SSSA.

Rice and Gavrilets (Rice et al., 2012) argued that a misexpressed epigenetic modifier of testosterone sensitivity or insensitivity that affected development of the brain only and not the body and genitals might possibly explain why homosexual people show SSSA but do not have intersex bodies. This is an interesting theory, but there is currently no evidence such a precise epigenetic modifier of testosterone sensitivity exists in either humans or other animals.

However, it is proposed, the endocrine hypothesis effectively categorizes homosexuals as partially intersex: homosexual men as partially feminized and homosexual women as partially masculinized (Mustanski et al., 2002; Balthazart, 2011). Such a portrayal of homosexuality perpetuates discredited ideas of homosexuality as sexual inversion (Ellis and Symonds, 1896), and the historic medical and psychological view of homosexuality as pathological. These views of homosexuality have long since been rejected by clinical and social psychology because in clinical psychology they have been found to be inaccurate, unsupported, and unconstructive (Haumann, 1995; Jordan-Young, 2010; Bailey et al., 2016). We argue that it is time for evolutionary psychology to also question the veracity of the endocrine hypothesis for human homosexuality.

Our proposed hypothesis for human SSSA has no requirement for sexual inversion. It would not require that SSSA be masculine-like for females or feminine-like for males. Rather, consideration of both an additive genetic model for SSSA and selection on SSSA in prosocial contexts would predict a diversity of expression of SSSA in both males and females.

We have argued that SSSA evolves as part of selection for increased prosociality. This hypothesis is testable. If it is correct there should be a detectable benefit to SSSA in contexts of within-group cohesion or cooperative tasks. Some evidence already points to a relationship between affiliation and SSSA in humans. Kirkpatrick (2000) documents ethnographic examples of SSSA and homosexual behavior strengthening important social affiliations in both males and females and SSSA supporting long term supportive social bonds. Human males self-reported a higher level of homoerotic motivation if they were primed with words related to friendship than if they were primed with words related to sex (Fleischman et al., 2015). This suggests that for males social affiliation is a greater releaser of SSSA than a sexual context (Fleischman et al., 2015). Whether within-group SSSA *enhances* cooperation and group performance to provide individual selective benefits remains to be tested, however.

Animal models could provide a powerful resource to explore these questions. We have described how homosexual behavior is more common in highly prosocial species than non-prosocial close relatives. We would predict homosexual behavior to enhance cooperation, group cohesion and performance and ultimately increase the reproductive success of individuals that are part of a high-functioning group in animals also. Comparing the consequences of homosexual behavior in bonobos and chimpanzees for group function would be a test of this hypothesis (Moscovice et al., 2019).

If the sociosexual hypothesis of SSSA evolution is correct we would expect to see an introgression of systems causal of human SSSA and social and affiliative behavior at both genetic and physiological levels of analysis. As we have discussed above, current evidence is compatible with this hypothesis, but significant gaps remain in our understanding of the genomic and neurophysiological basis of human sexual orientation and much work remains to be done.

Exploration of human SSSA has thus far been dominated by assumptions that the trait must be maladaptive (Bell et al., 1981; King et al., 2005; Wrangham, 2019). It may be timely and beneficial to explore alternatives that consider the sociosexual adaptive functions of same sex attraction and sexual behavior, and the full spectra of expression of SSSA.

## Author Contributions

AB and BH conceptualized and wrote the manuscript.

## Conflict of Interest

The authors declare that the research was conducted in the absence of any commercial or financial relationships that could be construed as a potential conflict of interest.
